# Stem cells enhance their chondrogenic differentiation in hydrogels by mechanical tumbling

**DOI:** 10.7150/ijbs.118859

**Published:** 2025-09-06

**Authors:** Xuemiao Liu, Weiguo Zhang, Xing Wang

**Affiliations:** 1 First Affiliated Hospital of Dalian Medical University, Dalian 116001, China.; 2 Beijing National Laboratory for Molecular Sciences, Institute of Chemistry, Chinese Academy of Sciences, Beijing 100190, China.

Cartilage injury has increased social and economic burdens as a common joint disease, but because of the avascular and hard-to-self-repair nature of cartilage tissue, it has posed a clinical challenge for the replacement and reconstruction of damaged cartilage 1. This commentary elaborates on the findings of Ayushman et al. 2 who investigated the role of “cell tumbling” as the core to reveal a new mechanism for enhanced chondrogenic differentiation of stem cells through nuclear mechanotransduction in 3D hydrogels. This finding provides a new perspective on cell-microenvironment interactions in the field of biomechanics, with far-reaching implications for stem cell fate determination and tissue engineering applications.

Primarily, Ayushman et al. introduced a new concept of “cell tumbling” in cell mechanics research, and pioneers the rapid 3D movement pattern of stem cells in hydrogels. This is a departure from traditional studies, which have largely examined cell motility on timescales spanning hours to days 3, 4. The present work challenges this paradigm by correlating minute-scale motility dynamics with stem cell differentiation. Importantly, this study leverages PEG-based SGs, a uniquely designed material enabling dynamic matrix reorganization and amplifying the relevance of localized mechanotransduction. Notably, the original study primarily utilized PEG-based sliding hydrogels, which may not fully recapitulate the biochemical and structural complexity of natural extracellular matrices. For instance, natural hydrogels such as collagen or hyaluronic acid, which are abundant in native cartilage tissue, possess intrinsic bioactive motifs and degradation properties that could modulate cell-matrix interactions more physiologically. This material specificity limits the generalizability of the findings to in vivo environments. To broaden the applicability, future research could explore similar mechanotransduction phenomena in natural hydrogels or other material platforms that more closely mimic in vivo conditions.

Additionally, Ayushman et al. used real-time imaging, nuclear mechanical analysis, and cell differentiation indexes to verify the existence and biological impact of cell tumbling behavior. The authors meticulously demonstrate that cell tumbling is not merely a physical artifact but rather a biologically intrinsic response to the SG mechanical environment. This conclusion is supported by evidence of enhanced cytoskeletal and nuclear dynamics. While the experimental design is rigorous, it would be enriched by the inclusion of molecular-level analyses, such as time-series data on cytoskeleton remodeling and chromatin state alterations. For instance, the integration of advanced techniques like single-cell epigenetic sequencing could provide a deeper understanding of the dynamic interplay between nuclear mechanics and chromatin accessibility during differentiation.

Moreover, Lamin A/C, intermediate filament proteins from the nuclear lamina encoded by the LMNA gene, play a central role in mediating the mechanosignaling of cytoskeletal forces into nucleus 5. Ayushman et al. revealed the role of the nucleus as a “mechanosensor” during “cell tumbling” by examining nuclear structure and chromatin markers. However, nuclear mechanotransduction is a multilevel biological process that includes multiple levels of signaling from the extracellular matrix to the cytoskeleton to the nucleoskeleton and chromatin 6. The multilevel mechanotransduction chain of the cytoskeleton, nuclear membrane structural proteins, and chromatin has not been analyzed in depth in this study. In addition, the regulation of chromatin state involves a variety of epigenetic modification markers, and it is difficult to fully explain the complex epigenetic mechanism behind the cell fate change by the observation of H3K9me3 alone. For example, H3K27me3, a well-known repressive histone mark, may collaborate with H3K9me3 to silence lineage-inappropriate genes during chondrogenic differentiation, while histone acetylation (e.g., H3K27ac) could enhance the accessibility of chondrogenic gene promoters. Incorporating these markers would provide a more nuanced understanding of the epigenetic regulatory network driving stem cell fate determination. Future studies should incorporate additional markers, such as H3K27me3 and histone acetylation modifications, to elucidate the full spectrum of epigenetic mechanisms underlying stem cell fate decisions. Furthermore, the combination of high-resolution microscopy and single-cell epigenetic sequencing will be of great scientific value in revealing the multi-level structure of cellular mechanotransduction in a more systematic way.

There is significant heterogeneity among different stem cells, and different types of stem cells have significant differences in response to mechanical stimuli, such as NSCs and MSCs may exhibit different mechanical behaviors in different microenvironments^7^. Future validation of the “cell tumbling” behavior in other stem cell types would help to expand its general application in tissue engineering. In addition, PEG-based hydrogels were mainly used in the study, while natural hydrogels, which are closer to the in vivo environment, were not explored. This singularity in material selection limits the applicability of “cell tumbling” behavior in complex physiological environments.

The discovery of cell tumbling opens new avenues in biomechanics and tissue engineering. By modulating early-stage mechanical stimuli, researchers could potentially steer stem cell differentiation towards desired lineages, offering promising applications in regenerative medicine. However, several key questions remain unanswered:1) Temporal Dynamics: How do short-term mechanical stimulations translate into sustained differentiation effects? Longitudinal studies are needed to evaluate whether the transient tumbling behavior observed in this study has lasting impacts on tissue regeneration. To address this, future investigations should incorporate in vivo experiments, such as implanting the hydrogel-cell constructs into animal models of cartilage injury, to assess the long-term effects of cell tumbling on tissue regeneration over weeks or months. Such studies would provide critical sustainability indicators, including the persistence of chondrogenic markers and the structural integrity of the regenerated tissue. 2) Mechanistic Pathways: What are the exact molecular signaling pathways linking mechanical cues to epigenetic reprogramming? The role of signaling molecules such as cPLA2 and ARA could be explored in greater detail to harness their potential in cell engineering.3) Clinical Translation: How can cell tumbling be optimized for clinical use? The scalability and reproducibility of SG hydrogels in therapeutic contexts need to be addressed.

In summary, Ayushman et al. had made a pioneering contribution to the field of stem cell biology by identifying cell tumbling as a novel mechanobiological phenomenon. Their work underscored the significance of early-stage mechanical interactions in determining long-term cell fates. Despite its groundbreaking nature, the study left room for further exploration into the molecular intricacies and broader applicability of cell tumbling. By addressing these gaps, future research could unlock the full potential of this phenomenon, paving the way for transformative advancements in regenerative medicine.

## Abbreviations

3D: Three dimensional
PEG: Polyethylene glycol
SG: Sliding hydrogels
NSCs: Neural stem cells
MSCs: Muscle stem cells
cPLA2: Cytosolic phospholipase A2
ARA: Arachidonic acid
